# 2251: An estrogen receptor α (ERα)-BMPR2-apelin axis mediates 17β-estradiol’s protective effects on right ventricular function in experimental pulmonary hypertension (PH)

**DOI:** 10.1017/cts.2017.214

**Published:** 2018-05-10

**Authors:** Andrea Lee Frump, Margie Albrecht, Sandra Breuils-Bonnet, Bakhtiyor Yakubov, Mary Beth Brown, Steeve Provencher, Sebastien Bonnet, Tim Lahm

## Abstract

OBJECTIVES/SPECIFIC AIMS: Women with pulmonary arterial hypertension (PAH) exhibit superior right ventricular (RV) function and survival compared with men, a phenomenon attributed to poorly understood cardioprotective effects of 17β-estradiol (E2). We hypothesize that E2, through ERα, attenuates PH-induced RV dysfunction by upregulating the pro-contractile and pro-angiogenic peptide apelin. This ERα-mediated increase in apelin is mediated by the myocardial remodeling effector bone morphogenetic protein receptor 2 (BMPR2). METHODS/STUDY POPULATION: ERα, BMPR2, and apelin were measured (western blot) in RVs from patients with PAH-induced RV failure and in RV homogenates from male or female Sprague-Dawley rats with sugen/hypoxia (SuHx) or monocrotaline (MCT)-induced PH. H9c2 rat cardiomyoblasts and cardiac endothelial cells were stressed with TNF-α (10 ng/mL) or staurosporine (50 nM)±E2 (100 nM; 24 h). ERα-, BMPR2-, and apelin-dependence were evaluated by siRNA (5 pM). Downstream apelin target and pro-survival factor ERK1/2 expression was measured (western blot). *p*<0.05 by ANOVA was considered significant. RESULTS/ANTICIPATED RESULTS: ERα correlated positively with BMPR2 and apelin expression in SuHx-RVs and human RVs. Treatment of SuHx-PH rats with E2 or ERα agonist increased RV BMPR2 and apelin, whereas RV apelin was decreased in E2-treated hypoxic ERα knockout mice (*p*<0.05), but not in ERβ knockout mice. In H9c2 cells, E2 or ERα agonist attenuated TNF-α- or staurosporine-induced decreases in BMPR2, apelin, and phospho-ERK1/2 (*p*<0.05 for all endpoints). E2 protection was lost in presence of siRNA directed against ERα, BMPR2, or apelin (*p*<0.05). ERα was necessary for E2-mediated increases in BMPR2, apelin, and ERK1/2, and BMPR2 was required for the E2-mediated increase in apelin (*p*<0.05 for siRNA vs. scramble). ERα, BMPR2, and apelin protein was increased in decompensated human RVs but downstream phospho-ERK signaling was disrupted. DISCUSSION/SIGNIFICANCE OF IMPACT: E2, via ERα, increases BMPR2 and apelin in the failing RV and in stressed rat cardiomyoblasts. The E2-mediated increase in apelin is BMPR2-dependent and likely occurs through direct binding of ERα to the BMPR2 promoter. Harnessing this E2-ERα-BMPR2-apelin axis during RV compensation may lead to novel, RV-targeted therapies for PAH patients of either sex.

